# Genomic Analysis of a Highly Pathogenic Porcine Reproductive and Respiratory Syndrome Virus Circulating in Pig Farms in West China

**DOI:** 10.1128/genomeA.00507-18

**Published:** 2018-07-05

**Authors:** Chenyu Zhang, Hu Shan, Jianxin Wen

**Affiliations:** a College of Veterinary Medicine, Qingdao Agricultural University, Qingdao, China

## Abstract

Porcine reproductive and respiratory syndrome virus (PRRSV), which leads to tremendous economic losses worldwide, is currently one of the most threatening viruses for the swine industry. However, PRRSV outbreaks in West China are rarely reported, even though the virus has remained active for a long time across the country. In this study, we report an outbreak of the highly pathogenic PRRSV strain QTX, isolated from a pig farm located in Ningxia, a province in West China.

## Abstract (precis)

Porcine reproductive and respiratory syndrome virus (PRRSV), which leads to tremendous economic losses worldwide, is currently one of the most threatening viruses for the swine industry. However, PRRSV outbreaks in West China are rarely reported, even though the virus has remained active for a long time across the country.

## GENOME ANNOUNCEMENT

Porcine reproductive and respiratory syndrome (PRRS) is an acute contagious disease caused by PRRS virus (PRRSV), which is characterized by reproductive disorder, increased body temperature, and adverse respiratory symptoms (1). The first Chinese PRRSV strain, Ch-1a (2), was isolated from an aborted swine fetus from a pig farm in Beijing (3).

The genome of PRRSV is composed of a positive single-stranded RNA. The full-length genome sequence consists of about 15,000 bases (15 kb), which include open reading frame 1a (ORF1a), ORF1b, the GP2 to GP6 envelope proteins, the M matrix and nucleocapsid proteins, a typical 5′-end cap structure, and a 3′-end poly(A) tail (4, 5). ORF1a and ORF1b, accounting for about 80% of the viral genome, encode the replication enzyme of PRRSV (6).

The samples were thawed on ice and homogenized with a bead beater. Total RNA was extracted from homogenized tissue samples using TRIzol reagent following the manufacturer’s instructions. Reverse transcription (RT) of cDNA was performed with random hexamer primers on an RT system (Promega, Madison, WI, USA) according to the supplier’s instructions. The cDNA was then subjected to PCR amplification targeted to the GP5 gene to confirm the PRRSV infection. This viral strain was named QTX. Upon confirmation of PRRSV infection, a total of 23 pairs of primers covering the whole genome were identified among the highly conserved regions, according to a mass comparison of available genome sequences for PRRSV strains in GenBank. The whole genome of the QTX strain was amplified from the cDNA with these 23 pairs of conserved primers and cloned into the pEASY-T1 vector and sequenced by the Genwiz Company (Suzhou, China).

The replicase nonstructural protein 2 (Nsp2) of PRRSV is recognized as the most variable region within its genome. The Nsp2 gene can tolerate several mutations, insertions, and deletions. An isolate from the first outbreak of highly pathogenic PRRSV in China in 2006 was characterized by a discontinuous 30-amino acid [aa] deletion in the Nsp2 gene. Even though the 30-aa deletion is not related to the virulence of the virus, it has been used as an epidemiological genetic marker of highly pathogenic PRRSV since 2006 (7). After combing through the clinical symptoms and molecular evidence of Nsp2, we conclude that the PRRSV QTX strain isolated in this study belongs to the American-type highly pathogenic PRRSV.

The major envelope protein GP5 is often selected as one of the main targets for monitoring the genetic diversity of PRRSVs. GP5 is a glycosylated transmembrane protein, responsible for the attachment of the virus to the host cell and contains important immunological domains associated with virus neutralization (8). Nsp5 has a base deleted at position 245 and a base inserted at position 256, resulting in a 12-base frameshift between the two positions. In addition, mutations also occurred at the highly conserved base sites 7593, 7904, 10139, 13137, 13138, and 13168. These mutations may be related to local climate considerations, such as temperature and light intensity. These data will guide prevention and control strategies for PRRSV in pig farms located in West China.

### Accession number(s).

The complete genome sequence reported here was submitted to GenBank under the accession number KX357708.
